# Defining the flexibility window in ordered aluminosilicate zeolites

**DOI:** 10.1098/rsos.170757

**Published:** 2017-09-27

**Authors:** Stephen A. Wells, Ka Ming Leung, Peter P. Edwards, Matt G. Tucker, Asel Sartbaeva

**Affiliations:** 1 Department of Chemical Engineering, University of Bath, Bath BA2 7AY, UK; 2 Department of Chemistry, University of Bath, Bath BA2 7AY, UK; 3 Department of Chemistry, Inorganic Chemistry Laboratory, South Parks Road, Oxford OX1 3QR, UK; 4 Oak Ridge National Laboratory, Oak Ridge, TN 37831, USA

**Keywords:** zeolite, flexibility window, geometric simulation

## Abstract

The flexibility window in zeolites was originally identified using geometric simulation as a hypothetical property of SiO_2_ systems. The existence of the flexibility window in hypothetical structures may help us to identify those we might be able to synthesize in the future. We have previously found that the flexibility window in silicates is connected to phase transitions under pressure, structure amorphization and other physical behaviours and phenomena. We here extend the concept to ordered aluminosilicate systems using softer ‘bar’ constraints that permit additional flexibility around aluminium centres. Our experimental investigation of pressure-induced amorphization in sodalites is consistent with the results of our modelling. The softer constraints allow us to identify a flexibility window in the anomalous case of goosecreekite.

## 1. Introduction

The flexibility window [1] is a geometric property of zeolite frameworks [2–4], identifiable through template-based geometric simulation, e.g. using the GASP software [5,6]. This window is a range of framework densities within which the tetrahedra making up the framework can be made geometrically regular within a simplified, localized physical model.

Possession of a flexibility window is characteristic of extant zeolites, both natural and synthetic, but not of most hypothetical tetrahedral nets [7] proposed as potential zeolites, suggesting that the flexibility window is a requirement for hydrothermal synthesis of the framework. The flexibility window is also linked to pressure-induced phenomena including phase transitions and amorphization. Interestingly, the experimental densities of zeolites under ambient conditions are found to lie near the expanded edge of the flexibility window. From this point of view, zeolites can be viewed as a form of ‘expanded condensed matter’ [1,8,9].

The window as originally reported is defined for the framework modelled as pure silica, SiO_2_. The simulations are therefore aimed at giving all tetrahedra in the framework the same ideal geometry, with a Si–O bond length of 1.61 Å, and a O–Si–O angle of arccos(−1/3). The limits of the window are set both by the bonding topology of the framework and by steric exclusion of framework oxygen atoms, modelled as hard spheres with a contact radius of 1.35 Å. In a study on a siliceous faujasite structure, we have distinguished the intrinsic window, governed by the framework alone, from the extrinsic window controlled by interactions with extra-framework content inside small sodalite (SOD) cages [10]. Interestingly, we found that this is not always the case, as we have shown with crown ether and without in larger *t-wof* and *t-wou* cages in EMC-2 zeolite [11]. The geometric simulation approach is not limited to tetrahedral systems, and has, for example, been applied successfully to networks of regular and Jahn–Teller distorted octahedra in manganite perovskites [12–14].

In this study, we consider the geometry of aluminium and silicon tetrahedra in ordered aluminosilicate frameworks, using the SOD framework as a testbed. We explore the effect of modelling the AlO_4_ unit using either a strict tetrahedral constraint, or a softer ‘bar’ constraint which controls the Al–O bond distance but permits the O–Al–O bond to vary slightly. The ‘bar’ terminology indicates that the distance between a pair of atoms is constrained, as by a bar joining two spheres. The softer constraints provide a sensible definition of the flexibility window in the SOD and goosecreekite (GOO) frameworks with Al/Si ordering, and are consistent with our experimental investigation of pressure-induced amorphization in sodium sodalite and sodium bromide sodalite.

## 2. Results and discussion

**Figure 1.**
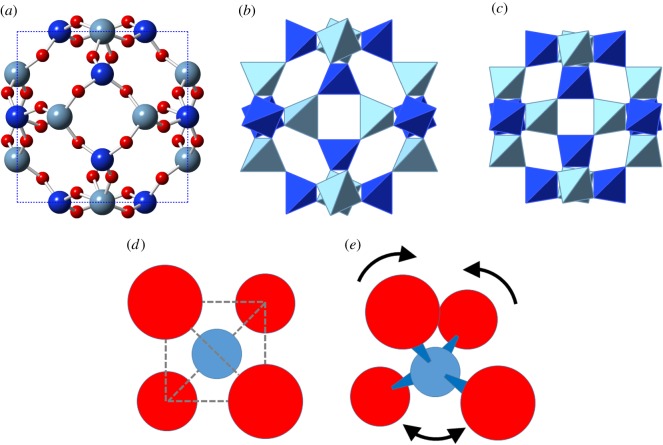
In the sodalite structure, the Si and Al tetrahedral centres lie on the faces of the unit cell (*a*) and define the characteristic *sod* or *β* cage. Dark blue— Si atoms/tetrahedra, light blue—Al atoms/tetrahedra and red—oxygen atoms. The structure is shown in the polyhedral view in (*b*). When the structure is compressed, the oxygen atoms of the four-rings move further out of plane so that each polyhedron rotates, as illustrated in the compressed structure in (*c*). Simulations using tetrahedral constraints (*d*) penalize any variation from ideal tetrahedral geometry. Simulations using bar constraints around Al centres (*e*) permit the bond angles to vary freely so long as the oxygen atoms do not collide with each other.

Mineral SOD is a fully ordered aluminosilicate framework with a framework density of approximately 16.7 tetrahedra per 1000 Å ^3^ [3]. However, a pure silica SOD has been produced by an unusual non-aqueous synthesis [15]. We, therefore, consider the flexibility windows of both purely siliceous and ordered aluminosilicate SOD frameworks. For a cubic SOD framework, the flexibility window is explored by varying the crystallographic *a* parameter alone. Every tetrahedral centre in a cubic SOD framework lies on a face of the unit cell (figure 1).

**Figure 2.**
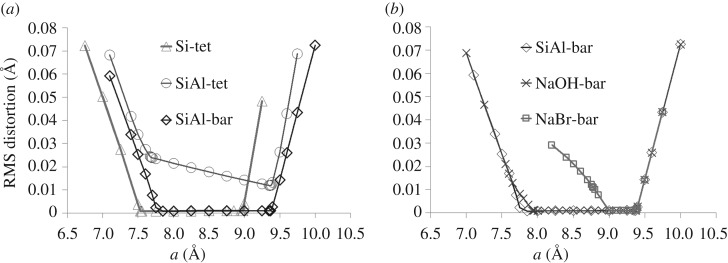
Polyhedral distortion. (*a*) Degree of polyhedral distortion (largest mismatch between the atomic position and the ideal vertex position) as a function of cell parameter for empty SOD framework modelled with Si tetrahedra (triangles); Si and Al tetrahedra (circles); and Si tetrahedra and Al-O bar constraints (diamonds). Lines are guides to the eye. (*b*) Degree of polyhedral distortion versus cell parameter for SOD framework modelled with Si tetrahedra and Al-O bar constraints, with the framework empty (diamonds) as before; with spheres representing Na content (crosses); with spheres representing Na and Br content (squares).

Modelling a pure-silica cubic SOD framework using GASP, we find a broad flexibility window. The cell parameter at maximum expansion, *a*=8.98 Å , lies just above the experimental value (8.836 Å) under ambient conditions, as is typical for silica zeolites. In compression, steric contacts between codimeric oxygens first occur at *a*=7.85 Å , and distortion of the tetrahedra becomes inevitable on compression below *a*=7.53 Å , the window edge. Cell parameters quoted to two decimal places should be taken throughout as accurate to 0.01 Å. Distortion data illustrating this flexibility window are plotted in figure 2.

**Figure 3.**
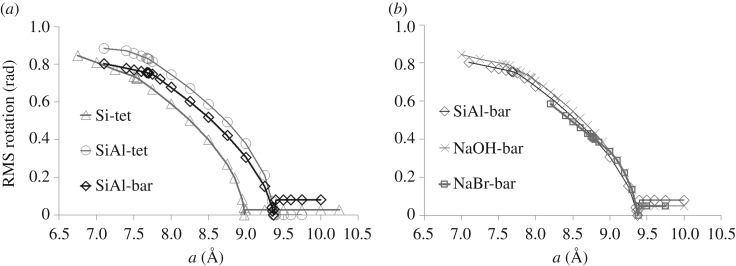
Tetrahedral rotation. (*a*) Degree of tetrahedral rotation of SiO_4_ units as a function of cell parameter for empty SOD framework modelled with Si tetrahedra (triangles); Si and Al tetrahedra (circles); and Si tetrahedra and Al-O bar constraints (diamonds). Lines are guides to the eye. (*b*) Degree of tetrahedral rotation of SiO_4_ units versus cell parameter for the SOD framework modelled with Si tetrahedra and Al-O bar constraints, with the framework empty (diamonds) as before; with spheres representing Na content (crosses); with spheres representing Na and Br content (squares).

The folding mechanism in compression involves counterrotation of adjacent tetrahedra (figure 1). The magnitude of tetrahedral rotation, measured relative to the maximally expanded structure at *a*=8.98 Å , is shown in figure 3*a*. The tetrahedral rotation is calculated by superposing corresponding tetrahedra and finding the best-fit rotation which minimizes their mismatch.

**Figure 4.**
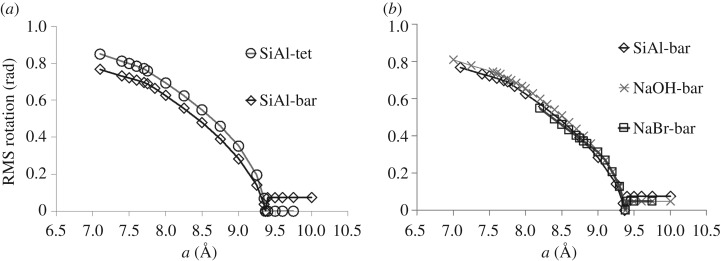
Tetrahedral rotation. (*a*) Degree of tetrahedral rotation of AlO_4_ units as a function of cell parameter for empty SOD framework modelled with Si and Al tetrahedra (circles); and Si tetrahedra and Al-O bar constraints (diamonds). Lines are guides to the eye. (*b*) Degree of tetrahedral rotation of AlO_4_ units versus cell parameter for the SOD framework modelled with Si tetrahedra and Al-O bar constraints, with the framework empty (diamonds) as before; with spheres representing Na content (crosses); with spheres representing Na and Br content (squares).

We now examine the fully ordered Si/Al SOD framework containing Al- and Si-centred tetrahedra. Initially, we define two forms of regular tetrahedral template, with ideal bond lengths of 1.61 Å for SiO_4_ and 1.75 Å for AlO_4_ units, an approach which was previously successful in investigating the pressure behaviour of wairakite [16,17]. However, this introduction of two differently sized tetrahedra in SOD causes intrinsic strain in the framework at all densities. A central low-strain region can be distinguished from high-strain regions in compression and extension, as illustrated in figure 2*a*. Within the central region, the distortion varies linearly with the *a* parameter, and is minimized by expansion to *a*=9.37 Å . In compression, the strain increases slowly until the region of *a*=7.70 Å , and then rises much more rapidly on further compression. The folding mechanism, of in-plane counterrotations of adjacent tetrahedra, retains the same character as in the hypothetical SiO_2_-SOD framework; however, the magnitude of the rotation is greater for the Si tetrahedra and less for the Al tetrahedra, in inverse proportion to their bond lengths. Rotations for the Si/Al framework cases are measured relative to the maximally expanded structure at *a*=9.37 Å and are illustrated in figures 3 and 4.

The ‘softer’, more ionic character of bonding at an Al centre can be reflected by relaxing the requirement of strict tetrahedral geometry. We apply ‘bar’ constraints (figure 1), which require an Al–O bond length of 1.75 Å but do not limit the O–Al–O angle. As the O–O inter-vertex distance in AlO_4_ is greater than the O–O contact distance of 2.70 Å, this permits some angular flexibility. With this approach, the ordered Si/Al SOD framework displays a true flexibility window analogous to that defined for SiO_2_ systems, as shown in figure 2. The limits of this window correspond to the central region in the fully tetrahedral case, with the limit of expansion occurring at *a*=9.38 Å and of compression at *a*=7.69 Å . As the distortions of the AlO_4_ tetrahedral geometry are small, we can still measure the degree of polyhedral rotation of the AlO_4_ units, as shown in figure 4.

The top of the window for the fully ordered Si/Al SOD framework lies at a considerably lower density (larger *a*) than the experimental structure under ambient conditions, or the expanded limit of the pure-silica hypothetical window. The fully ordered AlSiO_4_ system is thus not maximally expanded, but somewhat contracted from its hypothetical maximal volume, presumably due to attractive electrostatic interactions between framework [AlO_(1/2)4_]^−^ units and extra-framework cations, such as Na^+^. It is a striking coincidence that two competing effects—the expansion of the framework due to the longer Al–O bond length, and its contraction due to electrostatic interactions—almost exactly cancel each other, so that the experimental cell parameter of aluminosilicate SOD under ambient conditions lies close to the expanded edge of the flexibility window of SiO_2_-SOD.

The geometric simulation method does not currently account for electrostatic attraction but does allow for steric (contact repulsive) interactions between the framework and extra-framework atoms or molecules. We consider two cases: a framework populated by small spheres, representing a sodium SOD containing sodium and possibly hydroxide ions, and a framework including larger spheres representing a sodium bromide SOD. To model sodium SOD we begin with a crystal structure [18] with eight sodium sites in each cage. These sites each have 50% occupancy, corresponding to four sodium ions per cage, while the charge balance indicates the presence of additional, disordered hydroxide ions. We, therefore, populate a ‘bar’ constrained, fully ordered Si/Al framework with eight spheres, nominally ‘Na’ with a radius of 1.02 Å in each beta cage, to represent both the Na and OH content. The spheres are placed upon the eight crystallographic sodium sites in the cage. The resulting flexibility window is effectively identical to that of the empty fully ordered Si/Al framework (figure 2) and the inclusion of 8 extra-framework ‘Na’ atoms does not hinder the rotations of Si/Al tetrahedra (figures 3 and 4), with the rotation magnitudes being comparable to or even greater than those seen in the empty framework.

In the case of sodium bromide SOD [19], each cage contains four sodium sites and a central bromine site. In our modelling, we populate the cage with four ‘Na’ spheres and with a ‘Br’ sphere with radius 1.94 Å, placing these spheres on the sodium and bromine crystallographic sites. The point of maximum theoretical expansion, at *a*=9.37 Å , is identical to the framework with the presence of sodium only (figure 2*b*). However, the most compressed end of the flexibility window lies at *a*=9.00 Å , close to the experimental value. On further compression, the framework distortion increases rapidly. Figures 3*b* and 4*b* show the RMS polyhedral rotation of a bar-constrained, fully ordered Si/Al SOD framework with sodium and bromide ions. The effect of the large bromide ion in hindering the rotation of the polyhedra is visible in the decrease of the rotation magnitudes compared to the empty-framework case.

**Figure 5.**
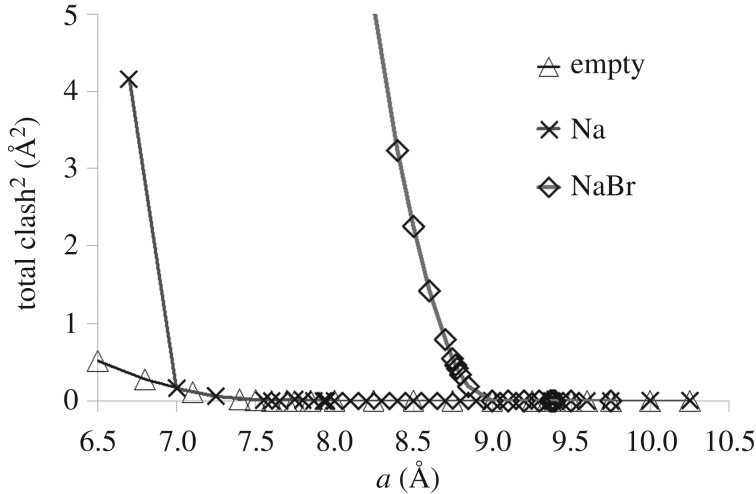
Degree of steric clash (total squared overlap of spheres) in the geometric simulations for the empty framework, the ‘Na’-loaded framework and the NaBr framework. Note the onset of substantial clashes in the NaBr framework from *a*≃9.00 Å and in the Na framework from *a*≃7.00 Å .

To measure the significance of steric contacts involving Br^−^, Na^+^ and framework O spheres, we track the sum of squares of steric overlaps, a ‘total clash^2^’ measure. Figure 5 shows the ‘total clash^2^’ of empty, sodium and sodium bromide bar-constrained, fully ordered Si/Al SOD frameworks. With no extra framework contents, no steric contacts are observed until *a*=7.85 Å . Clashes between framework O atoms start to build up from around *a*=7.75 Å . In the presence of 8 ‘Na’ spheres, clashes between ‘Na’ and framework O are first observed at *a*=8.90 Å , but these can be resolved. Clashes among ‘Na’ spheres occur from *a*=6.94 Å causing a rapid increase in the clash measure. When the framework contains large ‘Br’ spheres, steric clashes become significant and lead to polyhedral distortion as soon as the ambient-pressure structure is compressed.

**Figure 6.**
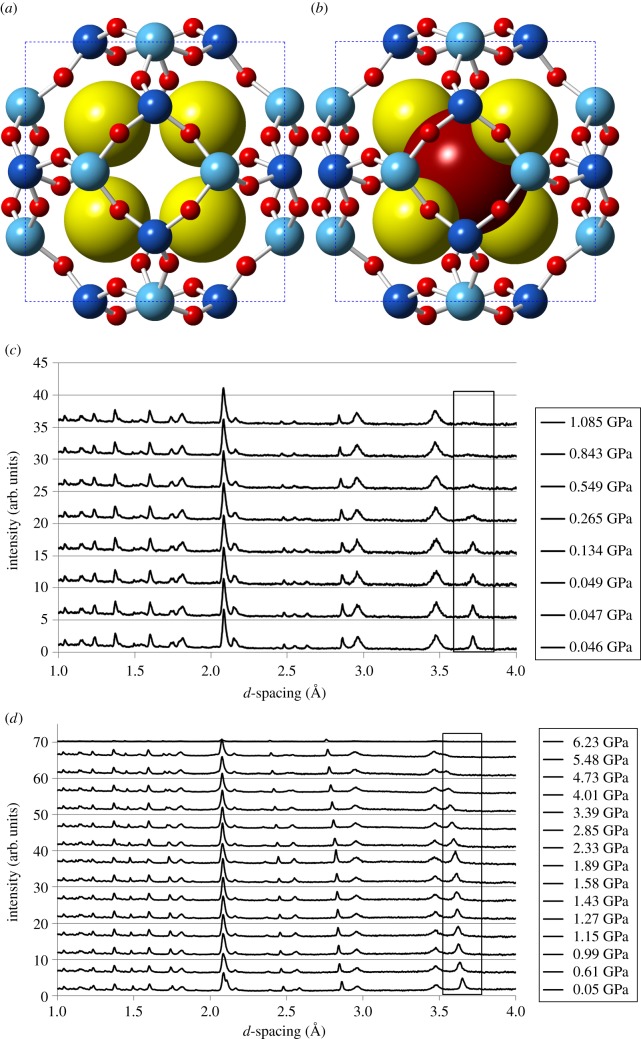
Sodium sites in Na-Sod (*a*) and sodium and bromine sites in NaBr-Sod (*b*) are shown as larger spheres. Experimental powder diffraction data on sodalites (*c*) Na-Sod and (*d*) NaBr-Sod. Box outlines the sodalite (211) peak; other peaks are attributable to the sample holder and the pressure standard (lead, alumina and zirconia). In Na-Sod, amorphization is seen over the range 0.2–1.0 GPa, while NaBr-Sod is crystalline to above 4 GPa.

Previous comparisons of geometric simulations with compression data [9] on siliceous zeolites suggest that pressure-induced amorphization of a zeolite framework proceeds while the structure is *within* its flexibility window. It thus occurs at low pressures in an empty framework, whereas a structure stressed by the presence of bulky extra-framework content resists amorphization and retains crystallinity to higher pressures. We have therefore investigated the pressure-induced amorphization of sodium sodalite (Na-Sod), with the chemical formula Na_8_(Al_6_Si_6_O_24_)(OH)_2_, and sodium bromide sodalite (NaBr-Sod), Na_8_(Al_6_Si_6_O_24_)Br_2_. Both zeolites were synthesized hydrothermally by conventional methods [18,19] and subjected to compression in a Paris-Edinburgh cell at PEARL beamline at ISIS. The positions in the sodalite cage of sodium sites in Na-Sod and of sodium and bromine sites for NaBr-Sod are shown in figure 6*a*,*b*. We have used both Fluorinert and methanol/ethanol/water mixture as pressure-transmitting fluids, obtaining consistent results. The ambient lattice parameters are *a*=8.94 Å for NaBr-Sod, and *a*=9.02 Å for Na-Sod, in methanol/ethanol/water medium. Powder diffraction patterns are shown in figure 6*c* for Na-Sod and figure 6*d* for NaBr-Sod. The range of *d*-spacings accessible on PEARL allow us to compare the intensity of the sodalite (211) peak, observable at about 3.6 Å, to peaks attributable to the sample holder (alumina and zirconia) and the lead pressure standard; the sodalite peak is highlighted with a box in the figures. Na-Sod amorphizes rapidly under compression and loses crystallinity at pressures of 0.2–1.0 GPa. NaBr-Sod, by contrast, resists amorphization and retains crystallinity up to at least 4 GPa. This is consistent with our simulations using Al-O bar constraints, with Na-Sod displaying a broad flexibility window while NaBr-Sod is strained.

**Figure 7.**
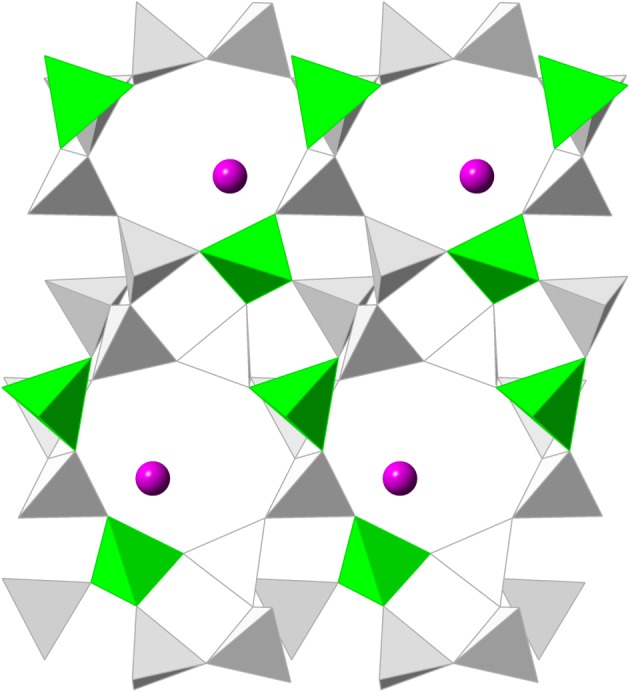
Goosecreekite structure. Spheres represent extra-framework calcium ions; the framework tetrahedra nearest to these ions are aluminium sites.

We now apply the Al-O bar constraint model to the case of GOO framework. This intriguing mineral is a relatively dense small-pore zeolite containing divalent Ca^2+^ cations. Although its Si:Al ratio is 3:1, the Al framework content is well localized to specific sites adjacent to the extra-framework cations. In the recent study of zeolite flexibility windows by Kapko *et al.* [20] GOO was a unique case in which the framework does not appear to display a flexibility window when modelled as a hypothetical pure-silica zeolite. Subsequent doctoral work by Dawson [21] reports that the framework can be relaxed using larger Al tetrahedra and lower symmetry, but does not report the cell parameter extent of the flexibility window. The structure of GOO as reported by Rouse & Peacor [22] is shown in figure 7.

We have carried out geometric simulations on GOO using three different models: the hypothetical Si-GOO case in which all tetrahedra are uniformly sized with ideal SiO_4_ geometry; the ordered aluminosilicate case with AlO_4_ units modelled with larger tetrahedral constraints; and the ordered aluminosilicate case with Al-O bar constraints. In the first two cases, we do not find evidence of a flexibility window, confirming the finding of Kapko *et al.* With Al-O bar constraints, however, the structure successfully relaxes at the experimental ambient cell parameters and thus lies within its flexibility window. This confirms that the specific localized aluminium geometry in this framework, allowing a degree of tetrahedral deformation not permitted in a pure silicate zeolite, is essential for its formation. Experimentally, the structure is monoclinic; its flexibility window is thus, in principle, a four-dimensional shape. We have explored the range of geometries achievable by variation of each independent parameter separately. To ensure a large enough simulation cell, we make use of a ‘2 1 2’ supercell for our simulations, with parameters 2*a*=14.80 Å , *b*=17.44 Å , 2*c*=14.59 Å , *β*=105.44^°^. The resulting ranges are as follows: for 2*a*, 13.68 to 15.39 Å; for *b*, 16.13 to 18.17 Å; for 2*c*, 13.27 to 15.38 Å; for *β*, 101.5^°^ to 111.0^°^, to an accuracy of ±0.5^°^. These ranges are consistent with the observation on SOD that the aluminosilicate lies somewhat within the range of its flexibility window rather than being close to the limit of maximal expansion.

## 3. Conclusion

In summary, we have shown that Al-O bar constraints can be applied in geometric simulations to model zeolite frameworks with explicit Al/Si ordering. Our theoretical findings on SOD are consistent with our experimental data on pressure-induced amorphization. We confirm the significance of localized Al sites to the flexibility of GOO. This study stands alongside our recent investigation of flexibility and extra-framework content in faujasite [10], and our methodological extension of geometric simulation software to handle metalorganic frameworks [6], showing that the capabilities of geometric simulation for framework structures can be extended far beyond its original remit in modelling SiO_2_ systems.

## Data accessibility

All data (high-pressure data and simulations) used in this article are deposited in the repository at the University of Bath at doi:10.15125/BATH-00399. For the GASP code, contact Dr Stephen Wells at S.A.Wells@bath.ac.uk.
